# VEGF expression, microvessel density and dendritic cell decrease in thyroid cancer

**DOI:** 10.1080/13102818.2014.909151

**Published:** 2014-09-25

**Authors:** Maya Gulubova, Koni Ivanova, Julian Ananiev, Julieta Gerenova, Aleksandar Zdraveski, Hristo Stoyanov, Tatyana Vlaykova

**Affiliations:** ^a^ Department of General and Clinical Pathology, Medical Faculty, Trakia University, Stara Zagora, Bulgaria; ^b^ Department of Endocrinology, Medical Faculty, Trakia University, Stara Zagora, Bulgaria; ^c^ Department of General Surgery, Medical Faculty, Trakia University, Stara Zagora, Bulgaria; ^d^ Department of Chemistry and Biochemistry, Medical Faculty, Trakia University, Stara Zagora, Bulgaria

**Keywords:** VEGF, CD31, microvessel density, dendritic cell, thyroid cancer, prognosis

## Abstract

Thyroid cancer is one of the five most common cancers in the age between 20 and 50 years. Many factors including the potent angiogenic vascular endothelial growth factor (VEGF) and different dendritic cell types are known to be related to thyroid tumourogenesis. The study was performed to address the expression of VEGF and microvessel density in thyroid cancers and to evaluate the effect of VEGF expression in thyroid tumour cells on the dendritic cells. We investigated 65 patients with different types of thyroid carcinomas: papillary (PTC), oncocytic (OTC), follicular (FTC) and anaplastic (ATC), immunohistochemically with antibodies against VEGF, CD1a, CD83, S100 and CD31. Our results suggest that the expression of VEGF is significantly more often in PTC than ATC (92.3% vs. 60.0%, *p* = 0.025). The microvessel density marked with CD31 in the tumour border of PTC was significantly higher as compared to FTC (*p* = 0.039), but not to ATC and OTC (*p* = 0.337 and 0.134). We found that CD1a- and CD83-positive cells were dispersed with variable density and in OC CD31^+^ vessel numbers were positively correlated with CD83^+^ dendritic cells in tumour stroma (*R* = 0.847, *p* = 0.016). We did not find statistically significant associations of the survival of patients with PTC after the surgical therapy with VEGF expression and MVD. In conclusion we may state that VEGF expression in tumour cells of thyroid cancer can induce neovascularization and suppress dendritic cells.

## Introduction

Vascular endothelial growth factor (VEGF) is the major cytokine engaged in tumour angiogenesis. It is unique among angiogenic factors as it is mitogenic for vascular endothelial cells. In mammals, there are six known members of the VEGF family: VEGFA, VEGFB, VEGFC, VEGFD (or FIGF), placental growth factor (PlGF) and VEGFE (of viral origin).[1,2] The proliferative action of VEGF is predominantly restricted to endothelial cells although it is also mitogenic for lymphocytes and induces monocyte migration.[3] VEGF stimulates the *de novo* formation of blood vessels from vascular precursor cells and has several other pro-angiogenic activities such as induction of endothelial expression of proteases,[4] stimulation of microvascular leakage [5] and maintenance of continual survival of nascent endothelial cells.[6] Most tumour types overexpress VEGF mRNA. This expression directly correlates with regions of neoangiogenesis.[7]

It is well known that VEGF expression is up-regulated in thyroid malignancies from epithelial origin as compared to normal thyroid tissue.[2,8] Several studies have demonstrated neoangiogenesis in thyroid proliferative lesions.[9,10] There was no clear relationship between microvascular density (MVD) measurement and different epithelial thyroid cancer types.

It was reported that VEGF, produced by tumour cells can inhibit the functional maturation of dendritic cells from 34-precursors [11–13] by blocking NF-κB transcription.[14] It was shown that the reduction of or IL-6 release in the tumour microenvironment favours lymphocyte-dendritic cell anti-tumour response.[1,15] Even more, CD11c^+^ dendritic cell precursors were found to be localized next to the lumina of newly formed capillary structures indicating that these cells had contributed to the increased vascular density.[14] As far as we know the impact of VEGF on dendritic cells in thyroid cancer has not been well studied yet.

Our study was performed to address first the expression of VEGF and MVD in malignant epithelial thyroid cancers, i.e. papillary (PTC), folicular (FTC) and anaplastic (ATC). Second, we investigated the effect of VEGF overexpression in thyroid tumour cells on the immature (CD1a^+^) and mature (CD83^+^) dendritic cells.

## Materials and methods

### Patients

Specimens were obtained from 65 patients who underwent resection of thyroid cancer at the Department of Surgery, University Hospital ‘Prof. St. Kirkovich’, Medical Faculty, Trakia University, Stara Zagora, between 1996 and 2012. The patients comprised 13 males and 52 females, aged 22 to 81 years (mean 55.6 years), 10 of them were at an age of less than 45 years, and other 55 patients were over 45 years old (Table 1). No patient received anti-cancer treatment prior to surgery. According to tumour staging, patients were defined as follows: 61.5% (*n* = 40) at stage I; 15.4% (*n* = 10) at stage II; 20% (*n* = 13) at stage III; and 3.1% (*n* = 2) at stage IV (Table 1). Tumour grading and staging was performed according to the TNM Classification of Malignant Tumours 7th Edition, by UICC 2009.

**Table 1.** Demographic and clinical data of the patients with thyroid cancers.

Cases	Gender	Age	Histology	pT	Ly node metastasis	Distant metastasis	pTNM staging	Follow-up status
1	Female	69.0	PTC	T3	None	None	III	Deceased
2	Male	59.0	PTC	T2	Yes	None	III	Deceased
3	Female	67.0	PTC	T1	None	None	I	Deceased
4	Male	72.0	PTC	T2	Yes	None	III	Alive
5	Female	35.0	PTC	T1	None	None	I	Alive
6	Female	50.0	PTC	T1	None	None	I	Alive
7	Female	52.0	PTC	T3	None	None	III	Alive
8	Female	45.0	PTC	T1	None	None	I	Alive
9	Female	63.0	PTC	T1	None	None	I	Alive
10	Female		PTC	T1	None	None	I	Alive
11	Male	61.0	PTC	T1	None	None	I	Alive
12	Female	54.0	PTC	T1	None	None	I	Alive
13	Male	73.0	PTC	T1	None	None	I	Alive
14	Female	69.0	PTC	T1	None	None	I	Alive
15	Female	49.0	PTC	T3	None	None	III	Alive
16	Female	52.0	PTC	T1	None	None	I	Alive
17	Female	67.0	PTC	T2	None	None	II	Deceased
18	Female		PTC	T2	None	None	II	Alive
19	Male	56.0	PTC	T1	None	None	I	Deceased
20	Female	58.0	PTC	T1	None	None	II	Alive
21	Female	46.0	PTC	T1	None	None	II	Alive
22	Female	66.0	PTC	T2	None	None	II	Alive
23	Female	72.0	PTC	T1	None	None	I	Deceased
24	Female	63.0	PTC	T1	None	None	I	Alive
25	Female	50.0	PTC	T1	None	None	I	Alive
26	Female	81.0	PTC	T1	None	None	I	*No data*
27	Female	53.0	PTC	T2	None	None	II	Alive
28	Female	30.0	PTC	T1	None	None	I	Deceased
29	Female	57.0	PTC	T1	None	None	II	*No data*
30	Female	54.0	PTC	T1	None	None	I	Alive
31	Female		PTC	T1	None	None	I	Alive
32	Female	22.0	PTC	T1	None	None	I	Alive
33	Female		PTC	T1	None	None	I	Alive
34	Female	35.0	PTC	T1	None	None	I	Alive
35	Female	39.0	PTC	T1	None	None	II	Alive
36	Female	66.0	PTC	T1	None	None	II	Alive
37	Female	51.0	PTC	T1	None	None	I	Alive
38	Female	22.0	PTC	T1	None	None	I	Alive
39	Female	38.0	PTC	T1	None	None	I	Alive
40	Female	50.0	FTC	T2	None	None	I	Alive
41	Male		FTC	T2	None	None	I	*No data*
42	Male	69.0	FTC	T1	None	None	I	Alive
43	Female	50.0	FTC	T1	None	None	I	Alive
44	Female		FTC	T2	None	None	I	Alive
45	Female	64.0	FTC	T4	None	None	IV	Deceased
46	Female	45.0	FTC	T2	None	None	I	Alive
47	Male	62.0	FTC	T2	None	None	I	Alive
48	Female	47.0	ATC	T3	None	None	III	*No data*
49	Female	49.0	ATC	T3	None	None	III	*No data*
50	Male	63.0	ATC	T1	Yes	None	III	Alive
51	Female	64.0	ATC	T1	None	None	I	*No data*
52	Male	49.0	ATC	T2	None	None	II	Deceased
53	Female	78.0	ATC	T1	None	None	I	Deceased
54	Female	63.0	ATC	T3	None	None	I	Alive
55	Male	54.0	ATC	T3	None	None	III	*No data*
56	Female	48.0	ATC	T3	None	None	III	Alive
57	Female	79.0	ATC	T4	None	None	III	Deceased
58	Male	58.0	OTC	T2	None	None	I	Deceased
59	Female	58.0	OTC	T1	None	None	I	*No data*
60	Female	40.0	OTC	T1	None	None	I	Alive
61	Female	66.0	OTC	T1	None	None	I	*No data*
62	Female	56.0	OTC	T1	None	None	I	Alive
63	Male	67.0	OTC	T2	Yes	None	III	Deceased
64	Female	57.0	OTC	T2	None	Yes	IV	Alive
65	Female	51.0	OTC	T1	Yes	None	III	Deceased

Thirty nine patients (60%) were diagnosed with the papillary histologic tumour type (PTC), 8 patients (12.3%) had oncocytic type (OTC), 8 patients had follicular type (FTC) and the other 10 (15.4%) had anaplastic type (ATC) (Tables 1 and 2). Deeper invasion (higher pT staging) and advanced stages (stage III or IV) were more frequent events in ATC and OTC than in PTC and FTC (*p* < 0.0001 and *p* = 0.010, respectively) (Table 2). Tumour specimens were fixed in 10% buffered formalin and embedded in paraffin. Histological grading was performed on hematoxyllin and eosin-stained sections according to the protocols. Low differentiation and lack of tumour capsule was more often detected in specimens from ATC and OTC than from PTC and FTC (*p* < 0.0001 and *p* = 0.008, respectively) (Table 2).

**Table 2.** Demographic, clinical data and histological and pathological characteristics of the tumour specimens according to the thyroid tumour type.

Characteristics	PTC *N* (%)	FTC *N* (%)	ATC *N* (%)	OTC *N* (%)	*p*-value
Age (mean ± SD)	54.17 ± 14.48	56.67 ± 9.59	59.40 ± 12.05	56.62 ± 8.52	0.718*
Gender					0.316**
Males	5 (12.8)	3 (37.5)	3 (30.0)	2 (25.0)	
Females	34(87.2)	5 (62.5)	7 (70.0)	6 (75.0)	
pT classification					<0.0001**
T1	36 (92.3)	7 (87.5)	4 (40.0)	8 (100)	
T3	3 (7.7)	1 (12.5)	6 (60.0)	0 (0)	
Lymph node metastases					0.214**
No	37 (94.9)	8 (100)	9 (90)	6 (75.0)	
Yes	2 (5.1)	0 (0)	1 (10)	2 (25.0)	
Distant metastases					0.065**
No	39 (100)	8 (100)	10 (100)	7 (87.5)	
Yes	0 (0)	0 (0)	0 (0)	1 (12.5)	
pTNM staging					0.005**
I stage	25 (64.1)	7 (87.5)	3 (30)	5 (62.5)	
II stage	9 (23.1)	0 (0)	1 (10)	0 (0)	
III stage	3 (12.8)	0 (0)	6 (60)	2 (25.0)	
IV stage	0 (0)	1 (12.5)	0 (0)	1 (12.5)	
pTNM staging					0.010**
I–II stage	34 (87.2)	7 (87.5)	4 (40)	5 (62.5)	
III–IV stage	5 (12.8)	1 (12.5)	6 (60)	3 (37.5)	
Differentiation					<0.0001**
Low	3 (12.5)	0 (0)	3 (75.0)	3 (60.0)	
Moderate	19 (79.2)	1 (25.0)	1 (25.0)	2 (40.0)	
High	2 (8.2)	3 (75.0)	0 (0)	0 (0)	
Capsule					0.018**
None	4 (10.3)	2 (25.0)	5 (50.0)	4 (50.0)	
Intermediate	3 (7.7)	0 (0)	2 (20.0)	0 (0)	
Capsule	32 (82.1)	6 (75.0)	3 (30.0)	4 (40)	
Capsule					0.008**
None	7 (17.9)	2 (25.0)	7 (70.0)	4 (50.0)	
Present	32 (82.1)	6 (75.0)	3 (30.0)	4 (50.0)	

*ANOVA test.

**χ^2^ test.

### Immunohistochemistry

For immunohistochemical staining, the paraffin blocks were prepared using tumour tissues from the periphery of the tumour adjacent to the normal tissues. Paraffin sections 5 μm thick were dewaxed in two xyllenes (for 30 min each at 56 ºC) and were rehydrated in ethanol. Then the sections were soaked overnight in 10% sucrose in distilled water. Later, sections were washed in 0.1 M phosphate buffered saline (PBS), pH 7.4, incubated in 1.2% hydrogen peroxide in methanol for 30 min, and rinsed in 0.1 M PBS, pH 7.4, for 15 min. Then the slides were incubated in a humid chamber until night, at room temperature with antibody Monoclonal Mouse Anti-Human CD1a (DAKO, Denmark) and Anti-human S-100 protein (DAKO) in dilution 1:100, Monoclonal Mouse Anti-Human CD31, Endothelial Cell Monoclonal Antibody (DAKO) RTU, CD83 (Novocastra™, Leica Biosystem) in dilution 1:100, Monoclonal Mouse Anti-Human Vascular Endothelial Growth Factor (DAKO) in dilution 1:100. After washing three times with PBS, the slides were incubated with DAKO-REAL^TM^ En-Vision^TM^ detection system (DAKO) for 60 min, then visualized with diaminobenzidine and counterstained with Mayer's hematoxylin. PBS replacing the primary antibody was used as a negative control.

### Cell counting on immunohistochemistry

CD1a^+^, CD83^+^ and S-100^+^ cells were counted in the tumour stroma and at the tumour border, on five fields of vision in the areas with most intensive cell recruitment (hot spots) at a magnification (× 320, 0.74 mm^2^ area). In the surrounding normal thyroid the same cells were counted at five fields of vision, chosen at random, at a magnification (× 320, 0.74 mm^2^ area). The number of the positive cells was calculated at 1 mm^2^ area.

The microvessel density was assessed in at least three peritumoural areas (so-called ‘hot spots’) with the highest density of CD31. Positively stained isolated endothelial cells or cell clusters with or without visible lumina were counted as separate microvessels. The highest microvessel counts were used for statistical evaluation.

VEGF immunoreactivity was assessed semiquantitatively as ‘−’ for negative, ‘+’ for weak positivity, ‘++’ for strong positivity in tumour cell cytoplasm. For the statistical analysis cases were united as ‘−’ for negative and ‘+/++’ for positive.

### Statistical analysis

The SPSS 16.0 program for Windows was used for statistical analysis. The chi-squared test and Fisher's exact test were used to compare the immunohistochemical staining and the clinicopathological parameters. ANOVA, Student's *t*-test, Mann–Whitney *U* test and Kruskal–Wallis test were applied for comparing the continuous variables depending on the normality of the distribution. Correlations were tested by Spearmen and Person tests. Survival plots were drawn by the Kaplan–Meier test and survival periods were compared by log-rank test. The accepted level of significance was set at *p* < 0.05.

## Results and discussion

### Expression of VEGF in tumour tissue

**Figure 1.**
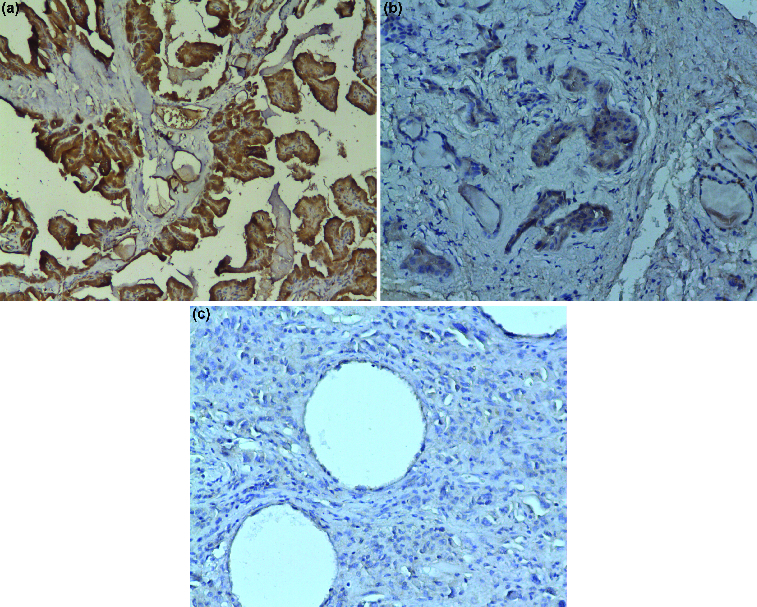
Vascular endothelial growth factor (VEGF) expression in thyroid tissue: (a) VEGF-positive immune reaction in follicular cells of papillary thyroid cancer (×100 magnification); (b) VEGF expression in follicular cells of follicular thyroid cancer (×200 magnification); (c) VEGF negative immune reaction in anaplastic thyroid cancer (×200 magnification).

In our study 65 patients were investigated immunohistochemically for VEGF in tumour cell cytoplasm (Figure 1(a)–(c)). Of them, 55 (84.6%) displayed expression of VEGF in tumour cell cytoplasm (Table 3). The comparison of the expression of VEGF between PTC and ATC showed a statistically significant difference in favour of PTC (92.3% vs. 60.0%, *p* = 0.025, Fisher's exact test) (Table 3).

**Table 3.** Immunohistochemical results for VEGF expression and S100- , CD1a- and CD83-positive cells in different types of thyroid cancers.

	PTC	FTC	ATC	OTC	
Pathological characteristics	Mean ± SD median (range)	Mean ± SD median (range)	Mean ± SD median (range)	Mean ± SD median (range)	*p*-values
S100-positive cells in tumour stroma	2.40 ± 2.18	4.62 ± 4.19	5.06 ± 5.27	5.33 ± 6.29	0.570**
	1.76 (0.68–8.65)	3.94 (0.81–9.11)	2.44 (0.68–13.74)	2.79 (1.08–14.69)	
S100-positive cells in tumour border	2.66 ± 2.35	4.98 ± 3.76	1.84 ± 1.10*	3.56 ± 2.34	0.433**
	2.58 (0–6.66)	3.53 (2.17–9.25)	1.76 (0.40–3.12)	3.44 (1.44–5.98)	
CD1a-positive cells in tumour stroma	6.61 ± 11.05	2.13 ± 3.11	1.58 ± 1.98	1.22 ± 0.58	0.190**
	1.76 (0–43.40)	1.08 (0.40–9.11)	0.76 (0–4.62)	1.63 (0.40–1.76)	
	*p = 0.085 vs. ATC				
CD1a-positive cells in tumour border	2.56 ± 3.86	1.31 ± 1.14	0.78 ± 0.89	0.62 ± 0.69	0.079**
	1.36 (0–20.13)	1.22 (0–3.53)	0.54 (0–2.31)	0.40 (0–2.04)	
	*p = 0.067 vs. ATC *p = 0.027 vs. OTC				
CD83-positive cells in tumour stroma	1.17 ± 2.67	0.43 ± 0.54	0.97 ± 0.81	1.18 ± 1.36	0.358**
	0.54 (0–11.15)	0.27 (0–1.49)	0.95 (0.2.31)	0.81 (0.13–4.21)	
		*p = 0.073 vs. OTC			
CD83-positive cells in tumour border	1.56 ± 3.32	0.50 ± 0.76	0.69 ± 0.52	1.18 ± 1.11	0.470**
	0.54 (0–16.59)	0.14 (0–1.90)	0.54 (0–1.49)	0.68 (0–3.12)	
VEGF					0.070^***^
Negative	3 (7.7%)	1 (12.5%)	4 (40%)	2 (25.0%)	
Positive	36 (92.3%)	7 (87.5%)	6 (60%)	6 (75.0%)	

*Mann–Whitney *U* test.

**Kruskal–Wallis test.

***χ^2^ test.

### CD31 staining

**Figure 2.**
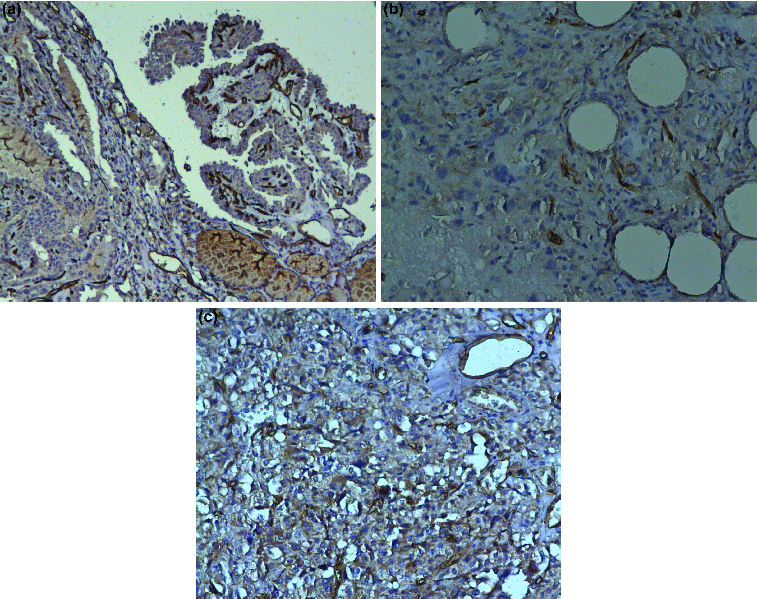
Microvessel density (MVD) in thyroid tissue: (a) CD31-positive vessels in a papillary thyroid cancer (×100 magnification); (b) CD31 staining in blood vessels in anaplastic thyroid cancer (×200 magnification); (c) CD31 staining in blood vessels in follicular thyroid cancer (×200 magnification).

**Figure 3.**
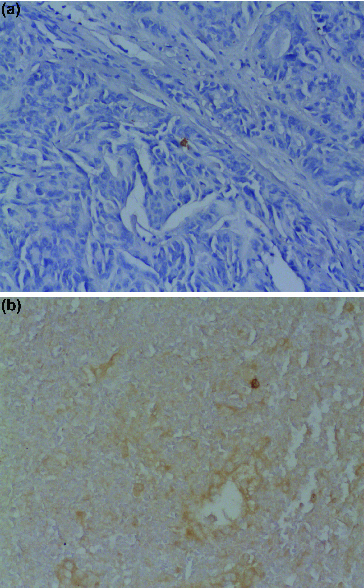
Blood vessel density in different types of thyroid cancers. Note: PTC, papillary thyroid cancer; FTC, follicular thyroid cancer; ATC, anaplastic thyroid cancer; OTC, oncocytic thyroid cancer. Data are presented as mean ± SD (*p = 0.039).

The immunohistochemically determined CD31-positive vessels (microvessel density, MVD) in the hot spots of tumour border were highly heterogeneous: they varied from 3.3 vessels per high power field (vessels/HPF) to 182 vessels/HPF (PTC, FTC and ATC, Figure 2(a)–(c)). The MVD in the tumour border of PTC was significantly higher (59.31 ± 42.79 vessels/HPF) as compared to FTC (28.35 ± 21.63 vessels/HPF, *p* = 0.039), but not to ATC (46.37 ± 21.11, *p* = 0.337) and OTC (37.04 ± 31.79, *p* = 0.134) (Figure 3).

The analysis of all tumour specimens, showed that tumours with capsule formation had significantly more CD31-positive vessels (59.36 ± 40.11 vessels/HPF) as compared to tumours without capsule (31.43 ± 27.16 vessels/HPF, *p* = 0.006). The documented difference was valid particularly for PTC: the mean value of MVD in PTC with capsule was 67.19 ± 42.42 vessels/HPF, while in PTC without capsule it was 23.28 ± 21.32 vessels/HPF (*p* = 0.012). We also observed that MVD was higher, although not significantly, in tumours with expression of VEGF in tumour cell cytoplasm (52.99 ± 40.31 vessels/HPF) compared to biopsies without detected VEGF expression (38.53 ± 26.01 vessels/HPF, *p* = 0.280, Student's *t*-test).

Unexpectedly, patients with advanced PTC cancers (stage III/IV) had tumours with lower levels of MVD (17.20 ± 14.26 vessels/HPF) compared to those with stage I/II (65.51 ± 42.14 vessels/HPF, *p* = 0.016, Student's *t*-test) and with lower expression of VEGF: 100% negative or weak VEGF expression in biopsies of stage III/IV and 59% of stage I/II (*p* = 0.139, Fisher's exact test).

### Dendritic cells

**Figure 4.**
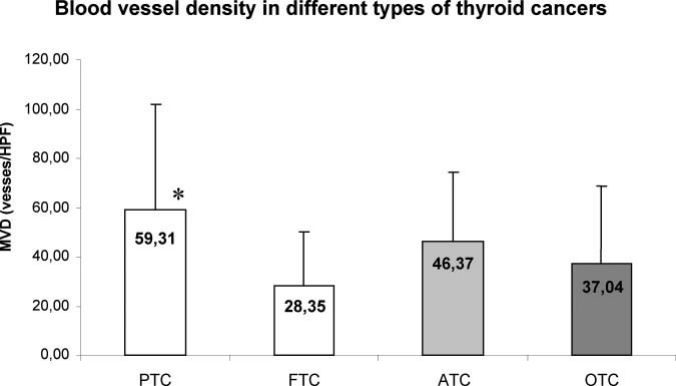
Dendritic cells in thyroid tissue: (a) CD1a-positive dendritic cells in papillary thyroid cancer (×200 magnification); (b) CD83-positive dendritic cells in anaplastic thyroid cancer (×200 magnification).

In the tumour stroma and border CD1a- and CD83-positive cells were dispersed with variable density (Figure 4(a) and 4(b)). For PTC CD1a-positive dendritic cells in tumour stroma were lower in number for VEGF positive cases compared to VEGF negative tumour cells (5.92 ± 10.43 vs. 13.05 ± 17.17 cells/mm^2^, *p* = 0.285, Mann–Whitney *U* test). Similarly, CD83-positive dendritic cells in tumour stroma were more in number when there was no VEGF expression in tumour cells (4.12 ± 6.12 vs. 1.48 ± 2.11 cells/mm^2^, *p* = 0.104).

**Figure 5.**
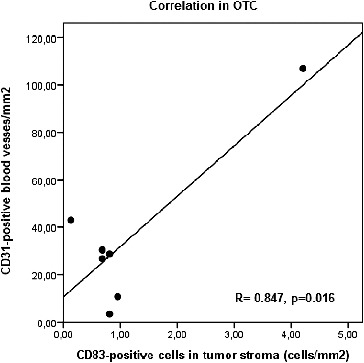
Correlation between CD1a- and CD83-positive dendritic cells in OTC (oncocytic thyroid cancer).

Vessel numbers in OTC CD31^+^ correlated positively with CD83^+^ dendritic cells in tumour stroma (*R* = 0.847, *p* = 0.016) (Figure 5), whereas in tumour border PTC CD31^+^ vessel numbers tended to correlate positively with S100^+^ dendritic cells (*R* = 0.438, *p* = 0.134).

### VEGF, MVD and survival of the patients

Complete clinical data including survival rates were available from the Oncological archives for 56 of the patients. These patients were followed-up until 30 April 2013. At the end of the follow-up period 42 patients were still alive: 30 of them with PTC (30/37), 6 with FTC (6/7), 3 with ATC (3/6) and 3 with OTC (3/6). The medial survival period for all patients was 104.41 months, ranging from 1.64 to 197.07 months. Patients with PTC had median survival of 104.27 months (12.76–182.28 months), those with FTC median of 123.38 months (18.15–197.08 months); with ATC median of 71.85 months (1.64–163.86 months), and patients with OTC median survival of 116.00 months (7.93–192.86 months).

**Figure 6.**
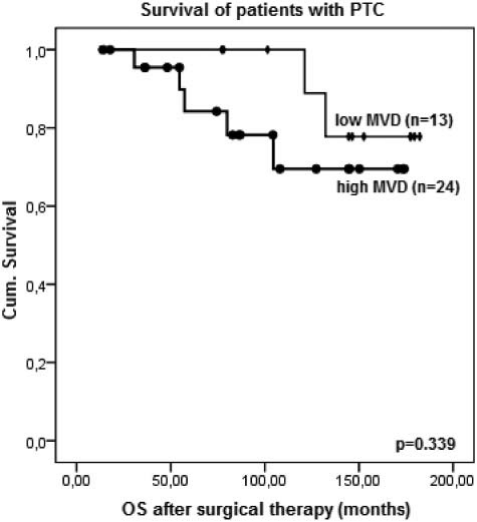
Kaplan–Meier survival plot for overall survival (OS) after surgical therapy of patients with PTC according to the microvessel density (MVD) (log-rank test).

Due to the very small number of patients with some types of thyroid cancer, we performed survival analyses only in the group of patients with PTC. A statistically significant association of the survival after the surgical therapy with VEGF expression and MVD was not determined. However, a particular trend was observed: patients with higher MVD (MVD higher than the median of 43.67 vessels/HPF) tended to have a worse prognosis than those with lower MVD (*p* = 0.339, log-rank test) (Figure 6).

VEGF produced from neoplastic cells is essential for tumour vasculogenesis, lymphocyte mitogenesis and monocyte recruitment [3] and for dendritic cell suppression.[2] The expression of VEGF in thyroid cancer has previously been demonstrated with *in vivo* and *in vitro* studies.[1,10,16–18] But still the role of VEGF is not fully understood. Some authors have reported that the higher VEGF immunostaining score correlated with the presence of lymph node or distant metastases in papillary thyroid cancer.[16] Others have shown that VEGF overexpression in tumour cells is connected with PTC size in adults and children.[19] Kilicarslan et al. [20] found an increased expression of VEGF in metastatic and recurrent disease in PTC. All cases in the present study did not show with metastases or recurrence in relation to the PTC, FTC and ATC types, except for a single patient with an OTC that had a distant metastasis.

The present report shows VEGF immune staining in thyroid tumour cells to be significantly more frequent in well-differentiated thyroid cancers like PTC as compared to poorly differentiated anaplastic cancers such as ATC. Higher VEGF expression in PTC suggests that VEGF plays an important role in early stages of carcinogenesis by supporting tumour growth through new vessels formation and also by exerting an immunosuppressive function. Probably, the lack of correlation between VEGF expression with clinical parameters like TNM, tumour stage, distant metastases and clinical outcome in our study is due to the fact that most of our patients were in early stages of the disease and in other reports, where half of the patients had metastases, VEGF overexpression correlated with metastasis occurrence and poor outcome.[16]

Our work also revealed significantly higher numbers of CD31^+^ vessels per HPF in peritumoural tissue in PTC as compared to FTC, and a tendency for increased MVD in PTC as compared to ATC. There was no statistical correlation between VEGF expression in tumour cells and MVD in all types of thyroid cancers. While there is a clear relationship between VEGF expression and differentiated PTC as compared to undifferentiated ATC, the data regarding the significance of MVD in thyroid neoplasms are rather confusing. Increased MVD was found in well-differentiated thyroid cancers and particularly in PTC by others.[2,21–23] Surprisingly, in some studies, reduced MVD in PTC was associated with advance stages, worse prognosis and reduced survival.[22,24] In contrast, in other studies, the poorer survival was connected to increased MVD.[25] Fontanini et al. [26] found an association of increased MVD with poor prognosis only for medullary, but not for papillary thyroid cancer. In our study, similar to the results of Frigugliettl et al., we detected that PTCs with advanced stage have lower MVD.[24] However, patients with PTC and lower MVD had a tendency for better survival. This can be explained with reduced ability for metastatic spread through vessels.

It is important to note that all studies about MVD in thyroid cancers employed different techniques to quantify MVD using CD31, CD34, etc. vascular markers, counted mainly in hot spots at higher magnification, where MVD was assessed as mean number at field of vision.[9,10,23] We also applied a method for evaluation of the MVD in the hot spots by measuring the CD31^+^ blood vessels per field of vision at higher magnification (HPF). Regardless of the differences in the assessment method, the estimation of MVD in epithelial thyroid cancers is necessary for better understanding of tumour behaviour. Further investigations could be held in order to reveal the impact of new stimulators of angiogenesis for thyroid cancer.

Dendritic cells present in tissues in an immature state and display low levels of maturation co-stimulatory molecules such as CD83, CD86 or CD80. Immature dendritic cells capture antigens including tumour antigens, undergo functional maturation process in response to IFN-α and present antigen, in a MHC class I or class II restricted manner to naive T cells. Mature dendritic cells secrete IL-12p70 and enhance killer cell activity and prime specific CD8^+^ T-cells that induce Th1 effective anti-tumour immune response.[13,15,27,28]

Dendritic cells in the present study were determined using monoclonal antibodies against CD1a and CD83 in order to mark immature and mature dendritic cells, respectively. We have found for the first time in thyroid cancer that the expression of VEGF was inversely related to the density of CD1a^+^ and CD83^+^ dendritic cells in tumour stroma, in PTC. The number of CD1a^+^ dendritic cells in VEGF-negative tumours was higher than that in VEGF-positive thyroid tumours, particularly in PTC. Similar inverse association was reported earlier for VEGF and S100^+^ dendritic cells in gastric cancer.[12] We previously have also reported such tendency in hepatocellular carcinoma.[29] The influence of VEGF on dendritic cells was revealed later as suppression upon dendritic cells’ ability to secrete IL-12p70 in response to lipopolysaccharide (LPS).[13] VEGF can elicit an inhibitory effect on differentiation and maturation on dendritic cells.[11,30,31] It was demonstrated that VEGF inhibited the expression of co-stimulatory molecule CD80 and the expression of CD54 (ICAM-1) on dendritic cells.[13] Moreover, VEGF has been shown to enhance in dendritic cells the phospho-ERK1 and ERK2, two MAP kinases involved in pathways that negatively regulate monocyte-derived dendritic cell maturation.[31] On the other hand, tumour-derived soluble VEGF can act as strong chemoattractant recruiting immature dendritic cells from the bone marrow precursors to tumour sites via chemokine receptor interactions.[28]

New investigations were held in recent years, showing that the same population of monocyte-derived dendritic cells could exhibit phenotype properties of mature dendritic cells under inflammatory conditions, or alternatively these dendritic cells can behave as endothelial-like cells in the angiogenic milleu.[14] Recently, it was shown that CD11c^+^ dendritic cells also expressed the vascular marker CD31 in an *in vivo* model to study the transdifferentiation of murine bone marrow-derived dendritic cells, into cells with markers, morphology and functional properties of endothelium.[32] We detected a positive correlation between CD31^+^ microvessels and the number of CD83^+^ and S100^+^ dendritic cells in the tumour stroma of OC and in the tumour border of PTC, respectively, facts that showed a connection between microvessel endothelium and dendritic cell accumulation at the same site. The concept that the formation of blood vessels by dendritic cells with endothelial potential was not restricted to animal models, but was also associated with angiogenesis in some human cancers, such as ovarian cancer.[33] All these data suggest that the tumour microenvironment can induce endothelization of dendritic cells.

Further investigations are necessary in order to reveal the role of dendritic cells in the tumour microenvironment of thyroid cancer.

## Conclusions

We may state that VEGF expression in tumour cells of thyroid cancer can induce neovascularization and suppression of dendritic cell recruitment in tumour stroma, and also may affect dendritic cell behaviour in neoangiogenesis.
